# Supplementary material for: Tissue composition shapes differential skeletal integration strategies during axolotl limb regeneration
Source: Nat Commun. 2026 Oct 15;17:10826. doi: 10.1038/s41467-026-78607-5 (PMC13672362; doi:10.1038/s41467-026-78607-5)
Supplement: Supplementary file 2 [file 41467_2026_78607_MOESM2_ESM.pdf]

nature portfolio

Peer Review File

Tissue composition shapes differential skeletal integration strategies during axolotl limb regeneration

Corresponding Author: Dr Tatiana Sandoval-Guzmán

This file contains all reviewer reports in order by version, followed by all author rebuttals in order by version.

Version 0:

Reviewer comments:

Reviewer #1

(Remarks to the Author)
This is an interesting paper from the group of Dr. Sandoval-Guzmán studying how skeletal degradation via osteoblasts is different depending upon whether the amputation is through the middle of the long bone (diaphysis) or at the end of the long bone (epiphysis). They use modern transgenic, imaging, and spatial transcriptomic approaches to address this interesting question. Overall, the manuscript is clearly written and easy to follow with very few mistakes. The findings will be of interest for the regeneration biology community, including mammalian to teleost fish. The sections that studying the differences in osteoclast activity are clear and convincing. The study looses focus once the bulk RNAseq analysis is introduced and the evidence for conclusions made for differences in muscle or epithelium based upon diaphysis versus epiphysis amputation planes is lacking.

-In the section titled: “Systemic and local calcium do not significantly impact osteoclast presence in regenerating limbs”, I am missing the context for why systemic or local calcium levels need to be ruled out. In either the introduction or in beginning of this section, some references for the calcium-based mechanism for osteoclast recruitment should be included.

Page 11, lines 258-266. Is there any indication of orthology based upon gene synteny? Is Loc483 surrounded by genes in syntenic regions in other species for either Ccl2, Ccl4, or Ccl24? Are there other genes in the axolotl assembly that align to these genes?

-In the section titled: “Diaphysis and epiphysis amputations differentially impact other processes related to regeneration”, it is unclear what the bulk RNA analysis is adding to the narrative. It seems reasonable to assume that the loss of spatial context in bulk versus spatial transcriptomics limits the resolution. The limited power of the bulk RNAseq with three limited biological replicates may make detection of subtle differences in a subset of cells hard to detect. I understand the motivation to include this data, but it doesn’t seem to be adding much to the study.

-Lines 302-309: Many of the gene expression changes between the two amputation planes are likely driven by the amount of each tissue type present in the bulk RNAseq sample. For example, the diaphysis muscle is likely due to the amputation taking place in the middle of the muscle, rather than the epiphysis.

-The last conclusion “The transcriptomic profile of the AEC is impacted by tissue composition at the amputation plane” is not as convincing as the first sections of the manuscript. There is a lot of speculation that leads to this conclusion and based upon the analysis, the evidence is not strong enough to definitely conclude that there is any physiologically important difference in the AEC between the epiphysis and diaphysis amputation. As stated, the reader would conclude that the data suggests the AEC has different functions based upon PD amputation planes, which has not been a common understanding in the field. Therefore, more conclusive evidence is needed for such a claim.

Reviewer #2

(Remarks to the Author)
Understanding the mechanisms of skeletal integration is critical for ensuring the structural and functional integrity of regenerated limbs. This manuscript addresses this fundamental problem by utilizing the axolotl limb as a unique model to investigate how the inherent heterogeneity of skeletal tissues influences regenerative strategies. The authors demonstrate that the tissue composition at the amputation plane exerts a decisive influence on osteoclast-mediated bone resorption, showing that amputation through calcified diaphysis triggers active resorption while amputation through cartilaginous epiphysis does not. Beyond this phenotypic divergence, the study identifies a novel chemokine Loc483/Ccl24-like which appears to play a critical role in promoting osteoclast recruitment and/or differentiation and further reveals that the transcriptomic signature of the AEC is significantly influenced by the tissue composition at the amputation plane. While these findings offer an intriguing conceptual framework, several major concerns regarding the experimental evidence and mechanistic interpretations must be addressed to support the primary conclusions. Specific points are as follows:
Major points:
1. The authors should better define the criteria used to distinguish tissue compositions at the two separate amputation planes (cartilage vs. bone). I suggest that the authors provide cross-sections at these two amputation planes and demonstrate the specific cellular and extracellular matrix (ECM) heterogeneity between calcified and cartilaginous regions.
2. In the calcium manipulation experiment, the authors only introduced extra calcium at the whole-animal or local levels. The rationale for performing this experiment should be provided. Moreover, the appropriate level of administered calcium is critical, as both excessively high and low levels may be ineffective. How was the positive readout of calcium administration demonstrated?
3. I am curious whether grafting calcified bone into a cartilaginous site would be sufficient to induce ectopic osteoclast recruitment.
4. Lines 156–158: The 3 dpa time point may not capture the early initiation phase. Please provide Ctsk/Ctsk-like expression data at an earlier stage (e.g., 1 or 2 dpa) to determine the precise onset of osteoclast recruitment and whether these precursors are already present at even earlier times.
5. Given the high sequence similarity, how does HCR distinguish between Ctsk and Ctsk-like transcripts? Please provide the detailed probe design strategy in the Materials and Methods.
6. In Figure 4, the expression patterns of ctsk and ctsk-like appear different. However, in Figure 2B, the HCR results show very good co-localization between these two genes. Considering my previous question, the authors should provide an explanation for this discrepancy.

Minor points:
1. Why is Loc483 spatially enriched in the same spots as Nfatc1 (a marker for osteoclast progenitors)? This spatial correlation requires a clearer explanation: is Loc483 expressed by the progenitors themselves or by the surrounding niche to recruit them?
2. In Figure 6F, I suggest the authors provide the experimental timeline for electroporation relative to amputation. Additionally, how was the overexpression of Loc483 validated? The authors should provide mRNA in situ hybridization evidence to confirm Loc483 overexpression.
3. I am curious about the potency of Loc483. Could it induce osteoclast recruitment in a non-injury/homeostatic environment (e.g., intact limb)? This would reveal whether Loc483 alone is sufficient to recruit osteoclasts or whether injury-induced co-factors are required.
4. Please provide correlation plots or PCA results for the three biological replicates of the bulk RNA-seq data to demonstrate the consistency and robustness of the transcriptomic findings.
5. For consistency across figures, I suggest swapping the positions of the diaphysis and epiphysis panels in Figure 1B (maintain a top-to-bottom or left-to-right convention in all figures).
6. In Figure 2B, there appears to be a labeling error in the color key for Ctsk and Ctsk-like; please verify and correct.
7. Lines 160–162: Clarify why Rank and Rankl appear in the same cluster. Typically, these represent a ligand-receptor pair derived from different cell types (e.g., osteoblasts vs. osteoclasts).
8. The findings reported in lines 284–287, stating “no difference” in bone- and osteoclast-related gene expression between diaphysis and epiphysis amputations based on bulk RNA-seq, require a more rigorous interpretation. Given that the authors identified differences in cellular composition and osteoclast recruitment at these two levels, does this lack of differential expression reflect true biological stability, or could it be a consequence of limited detection sensitivity in bulk-level analysis?

Reviewer #3

(Remarks to the Author)
The combination of transgenic reporter lines, in vivo live imaging, spatial transcriptomics, bulk RNA-seq, and functional gain-of-function experiments represents an elegant and technically impressive toolkit for addressing the study's central. This work provides several important observations of general interest. However, several aspects of this paper are mechanistically incomplete and require some revision to improve the manuscript.

1. Spatial transcriptomics resolution limitation not adequately addressed. First-generation 10X Visium captures mRNA at ~55 µm spot resolution, which is supra-cellular. Given the cellular heterogeneity of the axolotl limb on this scale, spot-level "clusters" likely represent mixtures of cell types. The authors acknowledge this briefly (line 201) but do not apply deconvolution (e.g., RCTD, Stereoscope, or cell2location) using a single-cell reference. Without deconvolution, assignment of the "resorption cluster" to osteoclasts specifically is circumstantial. This is especially important given the low frequency of osteoclasts in the tissue.
2. One of the major issues is that the statement “osteoclast-mediated skeletal resorption is primarily activated in amputations

damaging calcified regions of the skeleton, but not in cartilaginous areas". This could be misleading to many readers. Calcein will label mineralized cartilage. A lack of a distinction between mineralized cartilage vs ossification is problematic. The types of tissues present should be explicitly identified in both diaphyseal and epiphyseal locations and given the contextual link with embryonic mouse limb development (approximately E14-E15 in the animal sizes used in this manuscript. In mice Ossified bone develops at E15.5 and in the PIs previous paper (Camilo Riquelme-Guzmán et al 2022, PMID: 34322944) it states "Ossification was not detectable in ST 10cm animals. The first appearance of an ossified ring around the radius and ulna was observed in animals bigger than 12cm." There is another section that states "Epiphyses in the axolotl limb are mostly comprised of chondrocytes, which contribute to the growth of the limb and remain cartilaginous throughout the life of the animal7. In contrast, the diaphysis is where the primary ossification center first develops, which comprises a mix of hypertrophic chondrocytes, osteoblasts, and osteocytes, as well as periskeletal cells and a surrounding calcified extracellular matrix (ECM)7."

The only description that authors describe is that they used animals between "6-10cm" ST Length. Given the previous work it is important to describe the animal length within each experiment for interpretation. Additionally, animals can be stunted in size, but as they continue to age their immunological landscape will also change, which will alter the bone resorption kinetics. If the diaphysis cartilage cells are replaced by a primary ossification center at 12cm it would be important to check to see if this changes the OC program dynamics. Obviously with the expense of the spatial transcriptomics this may present challenges however this could be checked histologically and with HCR.

3. Spatial transcriptomics datasets are often biologically under replicated (frequently one section from one or two animals), making spot level signals vulnerable to section specific artifacts, spatial autocorrelation, and pseudo replication. Because each spot aggregates transcripts from ~10–30 cells, measurements represent binned mixtures rather than independent, cell resolved observations, lacking the variance structure and technical replication that underpin statistical inference in scRNA seq. As a result, spatial transcriptomics is best interpreted as hypothesis generating, with spatial patterns requiring validation across independent animals, sections, or orthogonal approaches rather than serving as standalone evidence of differential expression or cell states. Authors should confirm important spatially enriched genes using orthogonal, cell resolved methods such as HCR or immunohistochemistry, and integrating better with independent scRNA seq or snRNA seq datasets to demonstrate that spot level signals correspond to bona fide cell states rather than mixed or section specific effects. Where possible, concordance across modalities should be assessed at the pathway or gene set level rather than relying on single gene spot statistics.

4. In figure #4, in the 5dpa samples Epiph 1 is omitted where strangley in figure S3 in the 5dpa samples Epiph 2 is omitted. The authors should explain the exclusion criteria.

5. The authors demonstrate that Loc483 overexpression is sufficient to recruit osteoclasts in epiphysis amputations — a compelling result. However, the claim that it acts as a chemoattractant is not directly demonstrated. Specifically:
• No receptor identification or binding assay is presented
• No loss-of-functon(knockdown/knockout) experiment in the diaphysis condition is performed to show that Loc483 is necessary, not merely sufficient
• The authors should either perform LOF experiments or clearly demote this claim from "Loc483 is a chemokine" to "Loc483 is a candidate chemoattractant" throughout, including the title and abstract. Also, the manuscript alternates between using "Loc138491483/Ccl24-like," "Loc483," and "Ccl24-like" without a clear rationale for when each name is used. A single preferred name should be chosen upon first use and applied consistently. The authors' own phylogenetic analysis (Fig. S5) suggests the gene is likely not orthologous to human CCL24, making the "Ccl24-like" designation potentially misleading.

6. Number of independent experimental replicates is often unclear. For many HCR experiments, only "a minimum of 3 biological replicates" is stated. It would strengthen the study to report the exact N for each figure panel and to clarify whether experiments were independently repeated. e.g., for Figs. 2B, 5C, and 6E and S4, the exact N should appear in the figure legends.
7.

Other comments:
• Figures should be stand alone
• Figure 1- does not describe what colors are expected in this stain after EDTA decalcification.
• Figure S1- does not indicate the stain used?
• Epiphys pic does not look cut in fig 1 pic.
• Transgenic lines not mentioned in figure legends.
• Dual sox9 CTSK animals used with a mCherry reporter in limb electroporation?
• In Figure #3, how did the authors determine that these were the correct concentrations to use? Previously published or max safe tox limit?
• Animal age is not mentioned and may affect OC progenitors. Day 3 and 5 may not be appropriate for all sizes or ages. There is no justification to show that these timepoints optimal at this size.
• In fig 5A the authors should clarify if the data is derived from diaphysis, epiphysis amputations or both.
• FigS4C – the colors chosen Green and Magenta make white,and NFATc1 is also white. This needs to be broken up for clarity.
• Data deposition accession codes are missing. Both bulk RNA-seq and spatial transcriptomics raw data depositions list "GSEXXXXXX" as placeholders (lines 558–579). I am sure this will be corrected before publication.
Typos:
Line 642 contains "injected using using a fine heat-pulled glass capillary" — a typographical duplication that should be

corrected. Also note "capilary" is misspelled in line 643.
Line 126- “prevalence in the two amputations. Diaphysis amputations indeed showed robust osteoclast presence

Version 1:

Reviewer comments:

Reviewer #1

(Remarks to the Author)
The authors have thoroughly addressed the reviewers' comments, including new analyses and experiments that strengthen the support for their conclusions. They have also slightly modified the phrasing of some conclusions in response to concerns raised by reviewers. Overall, they have adequately addressed my concerns.

Reviewer #2

(Remarks to the Author)
The authors have thoroughly addressed all of my previous comments and made adequate modifications. I am delighted to recommend the current manuscript for publication.

Reviewer #3

(Remarks to the Author)
The authors have provided thorough and thoughtful responses to the reviewers' comments. The revised manuscript has been substantially improved and addresses most of the concerns raised during the initial review.

In particular, the authors have performed several elegant additional experiments that significantly strengthen the mechanistic conclusions of the study. The new data are of high quality, appropriately controlled, and provide mostly support the central claims. The revisions have also improved the clarity of the manuscript, with the rationale, results, and interpretation now presented in a much more accessible and convincing manner.

Although there are some minor subjective claims still present, I have no further substantive comments and believe the manuscript is now suitable for publication in its current form.

Open Access This Peer Review File is licensed under a Creative Commons Attribution 4.0 International License, which permits use, sharing, adaptation, distribution and reproduction in any medium or format, as long as you give appropriate credit to the original author(s) and the source, provide a link to the Creative Commons license, and indicate if changes were made.
In cases where reviewers are anonymous, credit should be given to 'Anonymous Referee' and the source.
The images or other third party material in this Peer Review File are included in the article’s Creative Commons license,

unless indicated otherwise in a credit line to the material. If material is not included in the article's Creative Commons license and your intended use is not permitted by statutory regulation or exceeds the permitted use, you will need to obtain permission directly from the copyright holder.
To view a copy of this license, visit https://creativecommons.org/licenses/by/4.0/

Point-by-point response to the reviewers

“

Reviewer #1 (Remarks to the Author)

This is an interesting paper from the group of Dr. Sandoval-Guzmán studying how skeletal degradation via osteoblasts is different depending upon whether the amputation is through the middle of the long bone (diaphysis) or at the end of the long bone (epiphysis). They use modern transgenic, imaging, and spatial transcriptomic approaches to address this interesting question. Overall, the manuscript is clearly written and easy to follow with very few mistakes. The findings will be of interest for the regeneration biology community, including mammalian to teleost fish. The sections that studying the differences in osteoclast activity are clear and convincing. The study loses focus once the bulk RNAseq analysis is introduced and the evidence for conclusions made for differences in muscle or epithelium based upon diaphysis versus epiphysis amputation planes is lacking.

1. In the section titled: “Systemic and local calcium do not significantly impact osteoclast presence in regenerating limbs”, I am missing the context for why systemic or local calcium levels need to be ruled out. In either the introduction or in beginning of this section, some references for the calcium-based mechanism for osteoclast recruitment should be included.

We thank the reviewer for pointing out that this section of the manuscript needed more context, which was also a point raised by Reviewer #2. We have now added an introductory paragraph to that section.

2. Page 11, lines 258-266. Is there any indication of orthology based upon gene synteny? Is Loc483 surrounded by genes in syntenic regions in other species for either Ccl2, Ccl4, or Ccl24? Are there other genes in the axolotl assembly that align to these genes?

By following the reviewer's suggestion of synteny examination to resolve the *Loc483* orthology, we have been able to uncover several interesting relationships between axolotl and human chemokines, and thus we thank them for their insight. Illustrations of these syntenic blocks have been added to the manuscript as Fig. 6g and Supplementary Fig. 6a.

The major findings include:

a) Synteny uncovered potential orthology between *Loc483* in the axolotl and several chemokine genes in other salamanders, frogs, and sharks based on being adjacent to the genes *Ppp2r3a* and *Epha3a-like*/*Epha4* (its exact name varying between species). Additionally, in one species of caecilian (representing the third major order of amphibians), two of the chemokines co-linear with *Epha3a-like*/*Epha4* were immediately upstream of the genes *Mff*, *Agfg1*, and *Daw1*. In amniotes, such as humans and chickens, these three genes are what surround the chemokine *Ccl20*, suggesting that *Ccl20* could be related to at least some of these amphibian and/or shark chemokines.
b) We did not observe any clear similarities in synteny between the genomic region containing *Loc483* in axolotl and the region containing *CCL24* in humans, which, along with *CCL26*, was surrounded by the genes *HIP1* and *RHBDD2* instead of *PPP2R3A*. Examination of *Hip1* in the axolotl found several other chemokines next to it, which was also the case in the caecilian and the coelacanth, the latter being a sarcopterygian that is an outgroup to all tetrapods. However, the exact relationship of these *Hip1*-adjacent chemokine genes to human *CCL24* is complicated by the fact that the newt, caecilian, and coelacanth genes also immediately flank the gene *Tmem132e*. In humans, this gene is also surrounded by two different stretches of multiple chemokines, one of which contains *CCL2* while the other contains *CCL4*. Regardless of the exact relationships of the axolotl's *Hip1*-adjacent chemokine genes to the human chemokines though, this synteny analysis reveals that *Loc483* is not likely to be closely related to human *CCL2*, *CCL4*, or *CCL24*.

We then tested these hypotheses through an expanded molecular phylogeny inference via maximum likelihood (ML) and Bayesian inference (BI) analyses using the amino acid sequences of all of these examined chemokines, supplied in Supplementary Data 11. The resulting phylogenetic trees have been added as Fig. 6f and Supplementary Fig. 6b. Both methodologies revealed that *Loc483* was part of a unique clade of chemokines, and that this clade has most likely existed since the last common ancestor of all jawed vertebrates. However, the members of this clade have since only been maintained in amphibians and sharks, with the most closely related gene remaining in mammals being *Ccl20*. As for the axolotl's *Hip1*-adjacent chemokines, both methodologies placed these genes within the clade containing human *CCL4*, although the exact relationships of all of the genes within this large clade differed between the ML and BI trees.

3. In the section titled: "Diaphysis and epiphysis amputations differentially impact other processes related to regeneration", it is unclear what the bulk RNA analysis is adding to the narrative. It seems reasonable to assume that the loss of spatial context in bulk versus spatial transcriptomics limits the resolution. The limited power of the bulk RNAseq with three limited biological replicates may make detection of subtle differences in a subset of cells hard to detect. I understand the motivation to include this data, but it doesn't seem to be adding much to the study.

The reviewer raises a relevant point that bulk RNA-seq has important limitations, crucially the loss of spatial resolution, as well as a lower power to detect differences in subsets of cells. Indeed, in this work, we have experienced exactly that by not detecting significant differences in expression levels of genes, such as *Ctsk* and *RANKL*, that we show to be differentially expressed by other methodologies. However, we still found that it was important to include this data to complement the spatial transcriptomics. As the latter technique can only examine the gene expression of one tissue section in one particular plane within the entire regenerating limb, the addition of bulk RNA-seq provides an essential whole-tissue overview of gene expression in diaphysis and epiphysis amputations. Importantly, by combining the spatial and bulk RNA-seq datasets, we were able to find that the AEC could be impacted by the tissues affected by the amputation (see answer to point 5 below)

However, we do agree that the aforementioned section could be improved. Thus, we decided to merge it with the next section of the manuscript and re-structure it based on this and the two comments below.

4. Lines 302-309: Many of the gene expression changes between the two amputation planes are likely driven by the amount of each tissue type present in the bulk RNAseq sample. For example, the diaphysis muscle is likely due to the amputation taking place in the middle of the muscle, rather than the epiphysis.

The reviewer raises a good point. Although we have strived to remove the same amount of tissue for bulk RNA-seq in the two amputation planes, it is true that the muscle content in the tissue varies in the diaphysis and epiphysis regions. To clarify this, we have added a new sentence emphasizing that this could be due to differences in tissue composition**.**

5. The last conclusion "The transcriptomic profile of the AEC is impacted by tissue composition at the amputation plane" is not as convincing as the first sections of the manuscript. There is a lot of speculation that leads to this conclusion and based upon the analysis, the evidence is not strong enough to definitely conclude that there is any physiologically important difference in the AEC between the epiphysis and diaphysis amputation. As stated, the reader would conclude that the data suggests the AEC has different functions based upon PD amputation planes, which has not been a common understanding in the field. Therefore, more conclusive evidence is needed for such a claim.

We agree with the reviewer that this part was not as strong as the rest of the manuscript. To improve it, we decided to test the hypothesis put forward in this section by investigating the expression pattern of the gene *AmexColB* by whole-mount in situ hybridization (WISH). This gene encodes a collagenase, and was found to be differentially upregulated in our bulk RNA-seq in epiphysis-amputated limbs (Supplementary Fig.10b, new Supplementary Fig.12b). Our stainings at 3 and 5 dpa confirmed that *AmexColB* was expressed in the AEC in both diaphysis- and epiphysis-amputated limbs, but also showed that its expression was stronger and more widespread in the latter (new Fig. 9d). This validation largely corroborated our conclusions that 1) the transcriptomic profile of the AEC differed between the two amputation planes; and 2) these could potentially translate into quantitative (amount of protein) and qualitative (expression pattern) differences in the secretory profile of the AEC.

Even though we have tested only one gene, these findings constitute proof-of-concept that the transcriptomic profile of the AEC is indeed impacted by tissue composition at the amputation plane. Ultimately, this suggests that these changes could potentially impact the physiology and functions of the AEC, which we had suggested before in one of our previous publications (Riquelme-Guzmán et al.,

2022). However, we agree with the reviewer that whether or not the transcriptional differences observed between the AECs result in significant functional consequences will require further investigation.

Finally, we apologize for the confusion that the use of the word "positional" potentially caused, as this indeed evokes PD positioning. Although tissue composition is ultimately determined by its PD position within the limb, our work focused solely on the impact of the former, and not of the latter, in the AEC profile or in the control of the resorption process.

Thus, we have restructured the entire final section of the paper, by merging the section "*Diaphysis and epiphysis amputations differentially impact other processes related to regeneration"* with *"The transcriptomic profile of the AEC is impacted by tissue composition at the amputation plane"*. We also included a new paragraph in this new part with the results of the *AmexColB* WISH, and have changed the wording in the conclusion of this section.

Reviewer #2 (Remarks to the Author)

Understanding the mechanisms of skeletal integration is critical for ensuring the structural and functional integrity of regenerated limbs. This manuscript addresses this fundamental problem by utilizing the axolotl limb as a unique model to investigate how the inherent heterogeneity of skeletal tissues influences regenerative strategies. The authors demonstrate that the tissue composition at the amputation plane exerts a decisive influence on osteoclast-mediated bone resorption, showing that amputation through calcified diaphysis triggers active resorption while amputation through cartilaginous epiphysis does not. Beyond this phenotypic divergence, the study identifies a novel chemokine Loc483/Ccl24-like which appears to play a critical role in promoting osteoclast recruitment and/or differentiation and further reveals that the transcriptomic signature of the AEC is significantly influenced by the tissue composition at the amputation plane. While these findings offer an intriguing conceptual framework, several major concerns regarding the experimental evidence and mechanistic interpretations must be addressed to support the primary conclusions. Specific points are as follows:

Major points:

1. The authors should better define the criteria used to distinguish tissue compositions at the two separate amputation planes (cartilage vs. bone). I suggest that the authors provide cross-sections at these two amputation planes and demonstrate the specific cellular and extracellular matrix (ECM) heterogeneity between calcified and cartilaginous regions.

We thank Reviewers #2 for this helpful comment, which is also echoed by Reviewer #3, as it made us realize that further clarification was necessary as to what diaphysis and epiphysis entails in the context of the axolotl skeleton. Thus, we have added a better description of the regions of interest in the first section of the main text, as well as high-magnification images of a typical skeletal element in the range of animal sizes used in our study (Fig. 1a) and a histological overview in a new supplementary figure (Supplementary Fig. 1a). However, we felt that longitudinal sections better conveyed the progressive differences in tissue heterogeneity between the two regions. This allowed us to clearly display differences in tissue composition in diaphysis and epiphysis regions, specifically by identifying hypertrophic chondrocytes (black arrow), periskeletal cells (white arrowheads), and mineralized ECM (black arrowhead) in the former, as well as chondrocytes in the latter (white arrow).

To keep the figure harmonious, we removed the schematic representation of the experimental set up. However, given its straightforward nature, we believe that this will not impair the readability or interpretation of the figure.

2. In the calcium manipulation experiment, the authors only introduced extra calcium at the whole-animal or local levels. The rationale for performing this experiment should be provided. Moreover, the appropriate level of administered calcium is critical, as both excessively high and low levels may be ineffective. How was the positive readout of calcium administration demonstrated?

We apologize for the omission of a proper context for this experiment, which Reviewer #1 also pointed out. We have now included a small paragraph regarding our motivations in the beginning of the section (see answer to point 1 of Reviewer #1 above).

In the experiments in which we manipulated extracellular calcium levels, this was done by injecting either BAPTA or $CaSO_4$ only locally, not systemically. Unfortunately, we could not find a way to

accurately monitor the effects of these local injections. Most available methodologies (such as calcium sensitive dyes) are developed for assessing calcium concentrations intracellularly, and assessing extracellular calcium concentrations frequently requires the usage of methodologies that are invasive, such as ion-specific probes. As such, the concentrations used in our experiments aimed at being as high as possible so that they could flood the tissue, but without compromising the overall regeneration of the limbs. This way, in our specific case, we took regeneration success as our readout that the compounds were administered successfully. However, we do acknowledge that our tests were mostly a first approach to this topic, and that calcium signaling during regeneration is a complex area that will require dedicated work in the future.

We have further clarified, both in the appropriate section of the Results and in the Methods, that BAPTA and $CaSO_4$ injections were performed locally in the regenerating limb tissue, and how the concentrations were chosen.

3. I am curious whether grafting calcified bone into a cartilaginous site would be sufficient to induce ectopic osteoclast recruitment.

Even though we share the reviewer's curiosity, this is a challenging experiment that would cause a significant amount of extra injury in the limb that could result in the resorption of the grafted tissue even without a subsequent amputation. For that reason, we predict that the results of this experiment would be difficult to interpret. However, we have devised an alternative experiment that we are confident will answer this question in a less invasive way.

For this, we took advantage of the existence of the two skeletal elements in the lower arm, the radius and the ulna, to perform staggered amputations that differentially affect each skeletal element. Specifically, we performed diaphysis amputations in the radius and epiphysis amputations in the ulna of the left limb, and the reverse amputations in the right limb of the same animal. We then analyzed the location of the $Ctsk^{+}$ signal in the limbs of Sox9::Ctsk animals. In these staggered amputations, we found that diaphysis-amputated limbs – regardless of it being the radius or the ulna – were able to induce osteoclast presence in the epiphysis-amputated skeletal element the majority of the times. This strongly suggests that calcified diaphysis amputations are indeed sufficient to induce ectopic osteoclast recruitment.

We have added these results in a new section of the paper entitled "*Diaphysis amputations can induce osteoclast recruitment in epiphysis-amputated skeletal elements*", and in a new Fig. 8.

4. Lines 156–158: The 3 dpa time point may not capture the early initiation phase. Please provide Ctsk/Ctsk-like expression data at an earlier stage (e.g., 1 or 2 dpa) to determine the precise onset of osteoclast recruitment and whether these precursors are already present at even earlier times.

We thank the reviewer for the suggestion. We have now included an HCR for Ctsk/Ctsk-like in a new supplementary figure (Supplementary Fig. 3), which shows that there are no apparent differences in osteoclast recruitment between the two amputation planes at 1 dpa. We have also added these results to its appropriate section in the main text.

5. Given the high sequence similarity, how does HCR distinguish between Ctsk and Ctsk-like transcripts? Please provide the detailed probe design strategy in the Materials and Methods.

The reviewer makes an excellent point, since, as we demonstrate by sequence alignment, the transcript sequences of Ctsk and Ctsk-like are quite similar. For this reason, we made sure to include the 5' and 3' UTRs to design the probes, which means that we used the entirety of the transcript for this purpose. The success of this probe design strategy is illustrated in Supplementary Fig. 3b. In this, we are able to detect subcellular differences in the localization of each transcript, which would not be apparent if the two probe sets would be detecting the same transcript.

We have updated the Methods section to better describe our probe design and validation strategies.

6. In Figure 4, the expression patterns of ctsk and ctsk-like appear different. However, in Figure 2B, the HCR results show very good co-localization between these two genes. Considering my previous question, the authors should provide an explanation for this discrepancy.

Having demonstrated in the previous point that the probe sets for *Ctsk* and *Ctsk-like* are specific, we believe that this discrepancy in expression patterns between HCR and spatial transcriptomics is caused by the imaging parameters used to acquire the HCR signal.

The expression of *Ctsk* and *Ctsk-like* in osteoclasts is very strong, which required imaging parameters that were optimized to avoid overexposure of this signal. This means that low-expressing cells will not be so evident in our images. Yet, these differences are still somewhat visible upon careful examination of Fig 2 and Supplementary Fig. 3, especially in the expression of *Ctsk* in epiphysis-amputated limbs.

However, in our spatial transcriptomics sets, that problem is avoided by displaying the gene expression in Log_2 scale. This way, we can detect that, despite their overlap, the expression pattern of the two genes is different, with *Ctsk* having a more widespread expression pattern than *Ctsk-like*.

Minor points:

7. Why is Loc483 spatially enriched in the same spots as Nfatc1 (a marker for osteoclast progenitors)? This spatial correlation requires a clearer explanation: is Loc483 expressed by the progenitors themselves or by the surrounding niche to recruit them?

The reviewer poses an interesting question. Indeed, those two explanations are possible, given that the resolution of our spatial transcriptomics is not subcellular. To try to answer this question, we used two approaches: 1) HCR stainings; and 2) analysis of previously published single-cell RNA-seq (scRNA-seq) of regenerating axolotl limbs (Li et al, 2021). We have included the results of these experiments in the main text and in a new supplementary figure (Supplementary Fig. 7).

Altogether, our experiments suggest that *Loc483*-expressing cells are not just one cell type with a defined role, but rather a diverse population, likely of immune cells, that includes osteoclast progenitors and differentiated osteoclasts, as well as surrounding non-osteoclast cells within the niche.. This data opens many new questions about the roles of these cells, which we are excited to one day tackle with the use of genetic cell tracing tools.

8. In Figure 6F, I suggest the authors provide the experimental timeline for electroporation relative to amputation. Additionally, how was the overexpression of Loc483 validated? The authors should provide mRNA in situ hybridization evidence to confirm Loc483 overexpression.

We thank the reviewer for the suggestions. Due to the addition of a phylogenetic tree for chemokines in Fig. 6, we have moved the gain-of-function for Loc483 to a new Fig. 7. In this, we included not only a timeline, but also a schematic representation for our electroporations. We have also added validation of the *Loc483* overexpression by including HCRs for this gene in two control and two experimental animals in Supplementary Fig. 8.

9. I am curious about the potency of Loc483. Could it induce osteoclast recruitment in a non-injury/homeostatic environment (e.g., intact limb)? This would reveal whether Loc483 alone is sufficient to recruit osteoclasts or whether injury-induced co-factors are required.

We thank the reviewer for the suggestion. Our data so far suggests that osteoclast recruitment during regeneration is a complex process that requires the presence of multiple injury-induced factors, such as RANKL, which are upregulated after amputation. Furthermore, given how intricate the regulation of osteoclast recruitment is in other animal models, we are certain that other injury factors not identified in this study will also play important roles in this process. For these reasons, our current hypothesis is that *Loc483* alone would not be sufficient to recruit osteoclasts in the context of an intact tissue due to the need of these other factors. Thus, though it is a very interesting experiment, we think that it is unnecessary in the context of this study.

10. Please provide correlation plots or PCA results for the three biological replicates of the bulk RNA-seq data to demonstrate the consistency and robustness of the transcriptomic findings.

We thank the reviewer for the comment. We have now included this information in a Supplementary Fig. 9.

11. For consistency across figures, I suggest swapping the positions of the diaphysis and epiphysis panels in Figure 1B (maintain a top-to-bottom or left-to-right convention in all figures).

We thank the reviewer for the suggestion. We have now made this change in Fig. 1b, in Fig. 9, and Supplementary Fig. 11.

12. In Figure 2B, there appears to be a labeling error in the color key for Ctsk and Ctsk-like; please verify and correct.

We thank the reviewer for pointing out this error. We have corrected it in Fig. 2b.

13. Lines 160–162: Clarify why Rank and Rankl appear in the same cluster. Typically, these represent a ligand-receptor pair derived from different cell types (e.g., osteoblasts vs. osteoclasts).

We have included a brief explanation regarding this point. We now explain that, altogether, our HCRs show that it is indeed different cells in the axolotl limb that are expressing RANK and RANKL, but these cells are found in close spatial proximity in the regenerating tissue, especially in diaphysis-amputated limbs. Considering the supracellular resolution of our spatial transcriptomics dataset, this explains our observation that RANKL and RANK are expressed in the same spatial cluster.

14. The findings reported in lines 284–287, stating "no difference" in bone- and osteoclast-related gene expression between diaphysis and epiphysis amputations based on bulk RNA-seq, require a more rigorous interpretation. Given that the authors identified differences in cellular composition and osteoclast recruitment at these two levels, does this lack of differential expression reflect true

biological stability, or could it be a consequence of limited detection sensitivity in bulk-level analysis?

We thank the reviewer for their closer examination of the seeming discrepancy between the bulk RNA-seq data and the apparent differences in cellular composition and osteoclast recruitment shown via HCRs and spatial transcriptomics, and agree that a more rigorous interpretation is warranted. However, we would like to clarify that we did not make the claim that there was "no difference" in bone and osteoclast related gene expression, only that any differences did not reach the statistical threshold necessary to be considered as differentially expressed. Based on the multiple methods we used to compare diaphysis and epiphysis amputations, we think that this absence of differential expression in the bulk RNA-seq data is most likely not representative of a true biological consistency. However, we cannot fully rule out the possibility that total expression levels of these genes throughout the entire tissue are more stable than would be expected from the HCRs and spatial data. We would argue that the more likely reason for these differences is inherent to the techniques themselves, such as the limited detection sensitivity in bulk level analysis and the spatial transcriptomic data only being able to capture one specific plane of the limb rather than the complete tissue. We have thus updated the paragraph to better address these issues. We hope that this extended discussion provides a better explanation of the reason that could cause the discrepancies in the results of our different techniques.

Reviewer #3 (Remarks to the Author):

The combination of transgenic reporter lines, in vivo live imaging, spatial transcriptomics, bulk RNA-seq, and functional gain-of-function experiments represents an elegant and technically impressive toolkit for addressing the study's central. This work provides several important observations of general interest. However, several aspects of this paper are mechanistically incomplete and require some revision to improve the manuscript.

1. Spatial transcriptomics resolution limitation not adequately addressed. First-generation 10X Visium captures mRNA at ~55 µm spot resolution, which is supra-cellular. Given the cellular heterogeneity of the axolotl limb on this scale, spot-level "clusters" likely represent mixtures of cell types. The authors acknowledge this briefly (line 201) but do not apply deconvolution (e.g., RCTD, Stereoscope, or cell2location) using a single-cell reference. Without deconvolution, assignment of the "resorption cluster" to osteoclasts specifically is circumstantial. This is especially important given the low frequency of osteoclasts in the tissue.

We thank the reviewer for the suggestion. We have now performed deconvolution of our spatial transcriptomics using a previously published scRNA-seq dataset (https://doi.org/10.1007/s13238-020-00763-1) and have now included this data. This helped to confirm many of our observations, and specifically corroborated our annotation of the resorption cluster (cluster 2) as containing osteoclasts along with other types of immune cells (Supplementary Fig. 4d).

2. One of the major issues is that the statement "osteoclast-mediated skeletal resorption is primarily activated in amputations damaging calcified regions of the skeleton, but not in cartilaginous areas". This could be misleading to many readers. Calcein will label mineralized cartilage. A lack of a distinction between mineralized cartilage vs ossification is problematic. The types of tissues present should be explicitly identified in both diaphyseal and epiphyseal locations and given the contextual link with embryonic mouse limb development (approximately E14-E15 in the animal sizes used in this manuscript. In mice Ossified bone develops at E15.5 and in the PIs previous paper (Camilo Riquelme-Guzmán et al 2022, PMID: 34322944) it states "Ossification was not detectable in ST 10cm animals. The first appearance of an ossified ring around the radius and ulna was observed in animals bigger than 12cm." There is another section that states "Epiphyses in the axolotl limb are mostly comprised of chondrocytes, which contribute to the growth of the limb and remain cartilaginous throughout the life of the animal7. In contrast, the diaphysis is where the primary ossification center first develops, which comprises a mix of hypertrophic chondrocytes, osteoblasts, and osteocytes, as well as periskeletal cells and a surrounding calcified extracellular matrix (ECM)7." The only description that authors describe is that they used animals between "6-10cm" ST Length. Given the previous work it is important to describe the animal length within each experiment for interpretation. Additionally, animals can be stunted in size, but as they continue to age their immunological landscape will also change, which will alter the bone resorption kinetics. If the diaphysis cartilage cells are replaced by a primary ossification center at 12cm it would be important to check to see if this changes the OC program dynamics. Obviously with the expense of the spatial transcriptomics this may present challenges however this could be checked histologically and with HCR.

We thank the reviewer for highlighting the importance of a more thorough description of the tissues analyzed in this manuscript, which was also touched upon by Reviewer #2. We have now included more details in the main text and a full histological characterization of the exact skeletal tissues affected by the amputations in Fig. 1a, and within the whole-limb context in Supplementary Fig. 1 (see also the reply to Reviewer #2 point 1 above). In these images, we show the skeletal elements of animals from the same size range as the ones used in all of the experiments for this paper (6-10 cm ST), which demonstrates that they are clearly not yet ossified. This is because ossification, as defined by the progressive vascularization of the skeletal element, together with the deposition of cortical bone and formation of the marrow cavity, is only detected in the axolotl at approximately 12 cm ST (as the reviewer stated from Riquelme-Guzmán et al., 2021) (doi: 10.1002/dvdy.407). This can be further confirmed in our used animals by observing the diaphyseal hypertrophic chondrocytes in the H&E images of the spatial transcriptomic, as well as in all HCRs shown in this manuscript. The reviewer is right in that a breakdown of all animal sizes and age per experiment is important for the best interpretation of our results, and this can now be found in Supplementary Data 1.

Regarding animal growth and bone resorption kinetics, this was exactly the main motivation behind our 2021 study. Axolotl growth depends heavily on external factors such as temperature and feeding regime, which, in turn, will influence skeletal differentiation. Thus, in order to characterize skeletal resorption during regeneration (Riquelme-Guzmán et al., 2022) (doi: 10.7554/eLife.79966), our lab had to first describe animal growth specifically in our colony (Riquelme-Guzmán et al., 2021). Interestingly, even in the very standardized conditions of our colony, we still find some variation in animal size when grown in similar periods of time, as showcased in Supplementary Data 1.

Finally, the kinetics of osteoclast-mediated skeletal resorption in animals with fully ossified limb skeletons has been previously investigated in our lab's 2022 publication (Riquelme-Guzmán et al., 2022), specifically in Figure 5 – figure supplement 1. In this, qPCR for osteoclast markers and CT scans of regenerating limbs confirmed that bigger animals also exhibited significant resorption of ossified skeletal elements in a conserved, yet slightly extended, time window. Thus, these findings, together with the results of this manuscript, support the conclusion that osteoclast-mediated skeletal resorption is primarily activated in amputations damaging calcified regions of the skeleton – regardless of it being just mineralized cartilage or fully ossified bone. We have therefore made changes to highlight that the calcein is binding to mineralized cartilage in our animals, and thus that the induction of resorption does not require the skeleton to be ossified.

As for giving a contextual link to embryonic mouse limb development, such comparisons are quite difficult to make given how these two species differ in the way that their ossification progresses. Contrary to the mouse, axolotls have a slower continuous conversion of calcified surface cartilage to ossified bone that lacks a true bone marrow, while their epiphyses remain cartilaginous throughout their entire lives. We thus think it is better not to directly compare axolotl bone formation to that of mouse, as it could mislead readers into thinking that their ossification processes are more equivalent than they are.

3. Spatial transcriptomics datasets are often biologically under replicated (frequently one section from one or two animals), making spot level signals vulnerable to section specific artifacts, spatial autocorrelation, and pseudo replication. Because each spot aggregates transcripts from ~10–30 cells, measurements represent binned mixtures rather than independent, cell resolved observations, lacking the variance structure and technical replication that underpin statistical inference in scRNA seq. As a

result, spatial transcriptomics is best interpreted as hypothesis generating, with spatial patterns requiring validation across independent animals, sections, or orthogonal approaches rather than serving as standalone evidence of differential expression or cell states. Authors should confirm important spatially enriched genes using orthogonal, cell resolved methods such as HCR or immunohistochemistry, and integrating better with independent scRNA seq or snRNA seq datasets to demonstrate that spot level signals correspond to bona fide cell states rather than mixed or section specific effects. Where possible, concordance across modalities should be assessed at the pathway or gene set level rather than relying on single gene spot statistics.

The reviewer is correct insofar as each spot in the 10X Visium spatial transcriptomics used in this study aggregates signal from more than one cell, as the diameter of each spot is 55µm. However, this is less of a setback in the axolotl than in other systems, given the size of their cell nucleus and the low-density of the tissues at the analyzed stages. This is illustrated by the graphs below. In A we quantified cell and nucleus sizes in different cell types in axolotl limbs. In B, the number of nuclei (as proxy for cells) per spot in the AEC and mesenchyme regions are displayed, indicating that, on average, each spot contains only 4-5 cells in our samples. Interestingly, due to their considerable size, osteoclasts can easily occupy an entire spot.

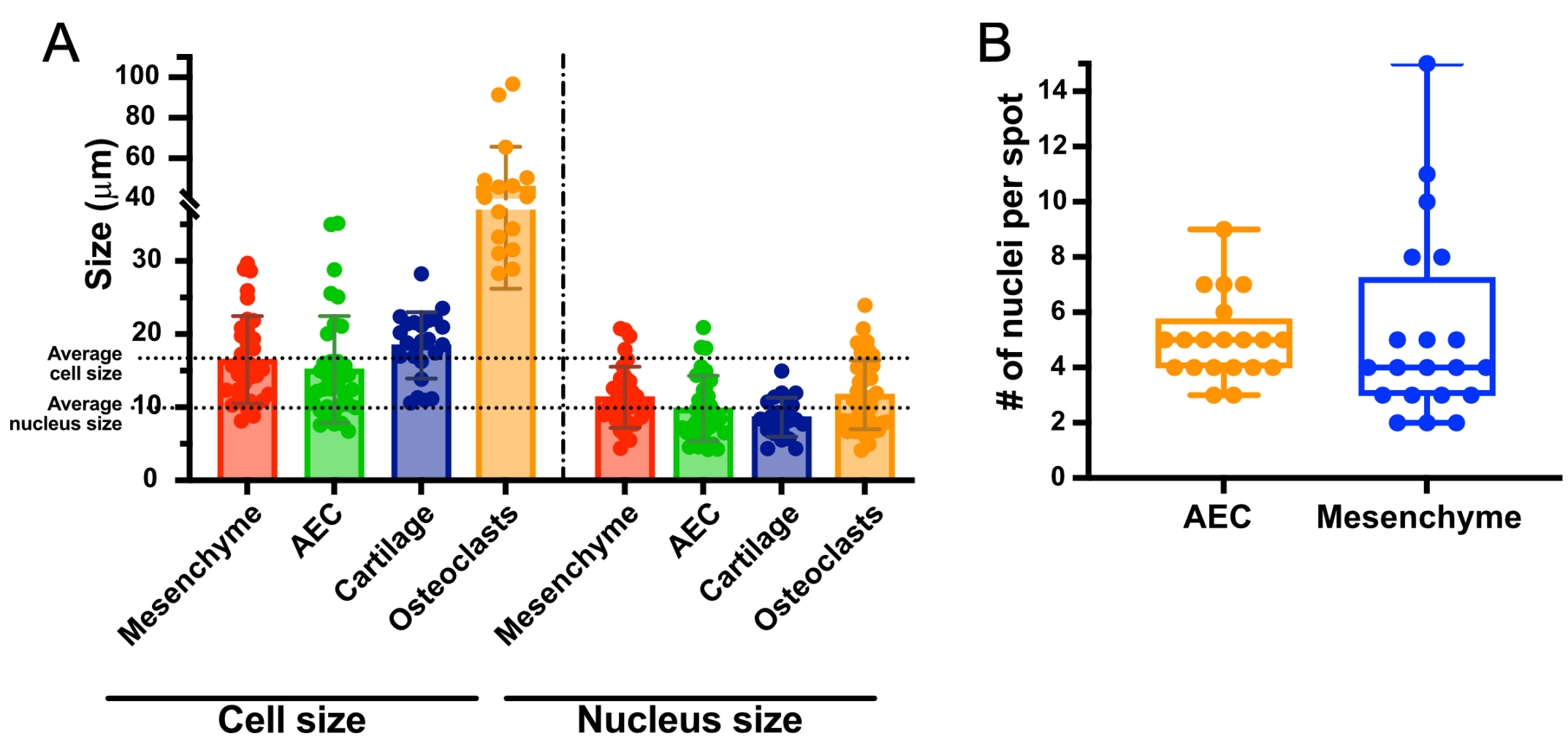

It was exactly due to the kinds of concerns the reviewer raises here that we made such efforts to validate all significant findings by means of HCR and WISH. Specifically, we have corroborated through these techniques that *Ctsk*, *Ctsk-like*, *RANK*, *RANKL*, *AmexColB*, and *Loc483* were differentially expressed reliably across independent animals, and in a reproducible manner. Furthermore, we have followed the reviewer's suggestion in point 1 and included deconvolution using the scRNA-seq (see above). This dataset was even used to answer questions about the identity of *Loc483* expressing cells (see answer to Reviewer #2, point 7 above, and the answer to point #5 below).

4. In figure #4, in the 5dpa samples Epiph 1 is omitted where strangley in figure S3 in the 5dpa samples Epiph 2 is omitted. The authors should explain the exclusion criteria.

We thank the reviewer for having detected this error. It has now been corrected to Epiph1 in Fig. 4 and Supplementary Figure 4.

5. The authors demonstrate that Loc483 overexpression is sufficient to recruit osteoclasts in epiphysis amputations — a compelling result. However, the claim that it acts as a chemoattractant is not directly demonstrated. Specifically:
   • No receptor identification or binding assay is presented
   • No loss-of-function(knockdown/knockout) experiment in the diaphysis condition is performed to show that Loc483 is necessary, not merely sufficient

The authors should either perform LOF experiments or clearly demote this claim from "Loc483 is a chemokine" to "Loc483 is a candidate chemoattractant" throughout, including the title and abstract. Also, the manuscript alternates between using "Loc138491483/Ccl24-like," "Loc483," and "Ccl24-like" without a clear rationale for when each name is used. A single preferred name should be chosen upon first use and applied consistently. The authors' own phylogenetic analysis (Fig. S5) suggests the gene is likely not orthologous to human CCL24, making the "Ccl24-like" designation potentially misleading.

We thank the reviewer for the comment. We did try to knockdown Loc483 through the electroporation of a morpholino, but we did not detect any significant effect. This is probably because *Loc483*-expressing cells constitute a diverse population of cells likely composed primarily of immune cells. In fact, a deeper exploration of this cell population by deconvolution with a previously published scRNA-seq revealed that the *Loc483*$^{+}$ population contains a variety of cell types, which include osteoclast progenitors and differentiated osteoclasts (see answer to Review #2, point 7). This makes the devising of a robust genetic targeting strategy very challenging.

However, we do apologize for the confusion that the text might have caused. In it, we meant only that, due to Loc483 being annotated as a chemokine, it could <u>potentially</u> act as a chemoattractant like many chemokines do in other systems. Indeed, our newly performed and more extensive phylogenetic analysis confirms that Loc483 is a chemokine, which is most closely related to the CCL20 family. It also agrees with our first analysis by suggesting that *Loc483* has seemingly been lost in all vertebrate lineages, except for amphibians and sharks.

We have now changed this sentence to better convey this idea and avoid any further misunderstandings. We have also made the naming of Loc483 consistent throughout the manuscript.

6. Number of independent experimental replicates is often unclear. For many HCR experiments, only "a minimum of 3 biological replicates" is stated. It would strengthen the study to report the exact N for each figure panel and to clarify whether experiments were independently repeated. e.g., for Figs. 2B, 5C, and 6E and S4, the exact N should appear in the figure legends.

We thank the reviewer for the comment. The exact N and the number of independent repetitions for each figure panel have now been included in the corresponding figure legends.

<u>Other comments:</u>

1. Figures should be stand alone

We thank the reviewer for the comment. We have now changed our figures to improve their readability by changing colors, and including timelines and schemes to make our experimental set ups as clear as possible.

2. Figure 1- does not describe what colors are expected in this stain after EDTA decalcification.

We thank the reviewer for the suggestion. An explanation of the staining has now been included in the Fig. 1 legend and main text.

3. Figure S1- does not indicate the stain used?

We apologize for the omission. The staining used in this figure has now been included in the figure legend.

4. Epiphys pic does not look cut in fig 1 pic.

We apologize for the confusion. We have now changed Fig. 1 to make the amputations clearer.

5. Transgenic lines not mentioned in figure legends.

Transgenic lines used have now been included in all applicable figure legend.

6. Dual sox9 CTSK animals used with a mCherry reporter in limb electroporation?

The reviewer is correct in that we used a CAGGS-mCherry plasmid to assess electroporation efficiency in animals that already contained a mCherry transgene expressed in cartilage cells. The reason for this is that it was important that the mCherry plasmid could be detected in the visible spectrum. Critically, the strong fluorescence intensity of the CAGGS-mCherry plasmid made it very easy to distinguish between the signal of the endogenous transgene and the electroporated construct, and thus there is minimal concern that the fluorescence was confused between them.

7. In Figure #3, how did the authors determine that these were the correct concentrations to use? Previously published or max safe tox limit?

We thank the reviewer for pointing this omission, which was also raised by Reviewer #2. The reviewer can find an answer in our response to Reviewer#2, point 2.

8. Animal age is not mentioned and may affect OC progenitors. Day 3 and 5 may not be appropriate for all sizes or ages. There is no justification to show that these timepoints optimal at this size.

We have partially answered this in our response to point #2, and we have now included a new Supplementary Data 1 with the sizes and ages of the animals used in each experiment. Yet, in our experience, axolotl age is not a good metric of growth, as it is dependent on several external factors. This not only results in variations in animal sizes between different colonies, but also in small variations even in the same colony and subjected to the same husbandry conditions. Thus, it is the standard practice in the field to favor specifying the size of the animal, instead of its age.

In this study, we used animals ranging from 6-10cm ST, which corresponded to 3-5 months old. For these animal sizes and age ranges, we chose these two time points primarily based on our observations of the timing of osteoclast recruitment in the tissue. Specifically, stainings for *Ctsk* and *Ctsk-like* expression showed that 3 dpa was the first time point in which we could see differential osteoclast recruitment between diaphysis and epiphysis amputations, which has been further corroborated by the new staining provided as an answer to Reviewer #2, point 4. Additionally, we selected 5 dpa because it was the first time point in which we detected robust osteoclast presence before the beginning of extensive resorption. Thus, for the focus of this paper, and for these animal sizes and ages, we found 3 and 5 dpa to be optimal.

However, the reviewer is right that we did not explore other animal sizes in this work, so it is possible that these time points are not optimal for all cases. Yet, interestingly, our lab previously detected signs of

osteoclast-mediated bone resorption as early as 3 dpa in 16 cm (>1 year-old) animals (Riquelme-Guzmán et al, 2021; figure 5 – supplement 1). This indicates that the onset of resorption in larger animals is relatively conserved in time, at least until they reach 16 cm in length.

9. In fig 5A the authors should clarify if the data is derived from diaphysis, epiphysis amputations or both.

We have now clarified in the figure legend that the UMAP displayed Fig.5a is the same as in Fig. 4, and thus that it represents all timepoints and amputation levels.

10. FigS4C – the colors chosen Green and Magenta make white, and NFATc1 is also white. This needs to be broken up for clarity.

We thank the reviewer for having pointed this out. We have changed the color of Nfact1 to yellow to make the staining clearer.

11. Data deposition accession codes are missing. Both bulk RNA-seq and spatial transcriptomics raw data depositions list "GSEXXXXXX" as placeholders (lines 558–579). I am sure this will be corrected before publication.

We have now included the complete information on the accession number of our datasets in the Methods and in the Data Availability section.

<u>Typos:</u>

Line 642 contains "injected using **using** a fine heat-pulled glass capillary" — a typographical duplication that should be corrected.
Also note "**capilary**" is misspelled in line 643.
Line 126- "prevalence in the two amputations. Diaphysis amputations indeed showed robust osteoclast presence

We thank the reviewer for having spotted these typos. They have now been corrected.
