# Supplementary material for: Tissue composition shapes differential skeletal integration strategies during axolotl limb regeneration
Source: Nat Commun. 2026 Oct 15;17:10826. doi: 10.1038/s41467-026-78607-5 (PMC13672362; doi:10.1038/s41467-026-78607-5)
Supplement: Supplementary file 4 [file 41467_2026_78607_MOESM4_ESM.pdf]

Description of Additional Supplementary Files

Supplementary Data 1: Animal sizes and ages used in this work.

Supplementary Data 2: Differentially expressed marker genes for each spatial cluster.

Supplementary Data 3: Differentially expressed marker genes used for scRNA-seq cluster annotation.

Supplementary Data 4: Top differentially expressed genes in spatial spots expressing Nfatc1 Log2 Exp > 2.

Supplementary Data 5: Bulk RNAseq normalized gene counts (CPM) for intact epiphysis and diaphysis regions, and epiphysis- and diaphysisamputated limbs at 3 and 5 dpa.

Supplementary Data 6: Differentially expressed genes in epiphysis- and diaphysis-amputated limbs at 3 dpa found by bulk RNA-seq.

Supplementary Data 7: Differentially expressed genes in epiphysis- and diaphysis-amputated limbs at 5 dpa found by bulk RNA-seq.

Supplementary Data 8: Differentially expressed genes used for Gene Ontology analysis.

Supplementary Data 9: Gene Ontology analysis of differentially expressed genes in epiphysis- and diaphysis-amputated limbs at 3 and 5 dpa.

Supplementary Data 10: Transcript, primers, and HCR probe sequences.

Supplementary Data 11: Amino acid sequences of chemokines used for phylogenetic analysis.

Supplementary Data 12: Multiple sequence alignments of chemokines used for phylogenetic analysis.
